# Exploratory metabolomic analysis for characterizing the metabolic profile of the urinary bladder under estrogen deprivation

**DOI:** 10.3389/fendo.2024.1384115

**Published:** 2024-05-31

**Authors:** Wei Zhang, Qingbo Yang, Yingying Song, Wenheng Liu, Yao Li

**Affiliations:** ^1^ Department of Urology, The Affiliated Hospital of Qingdao University, Qingdao, China; ^2^ Department of Cardiology, The Affiliated Hospital of Qingdao University, Qingdao, China

**Keywords:** menopause, estrogen deprivation, bladder dysfunction, metabolomics, biomarkers, amino acid metabolism

## Abstract

**Background:**

Estrogen homeostasis is crucial for bladder function, and estrogen deprivation resulting from menopause, ovariectomy or ovarian dysfunction may lead to various bladder dysfunctions. However, the specific mechanisms are not fully understood.

**Methods:**

We simulated estrogen deprivation using a rat ovariectomy model and supplemented estrogen through subcutaneous injections. The metabolic characteristics of bladder tissue were analyzed using non-targeted metabolomics, followed by bioinformatics analysis to preliminarily reveal the association between estrogen deprivation and bladder function.

**Results:**

We successfully established a rat model with estrogen deprivation and, through multivariate analysis and validation, identified several promising biomarkers represented by 3, 5-tetradecadiencarnitine, lysoPC (15:0), and cortisol. Furthermore, we explored estrogen deprivation-related metabolic changes in the bladder primarily characterized by amino acid metabolism imbalance.

**Conclusion:**

This study, for the first time, depicts the metabolic landscape of bladder resulting from estrogen deprivation, providing an important experimental basis for future research on bladder dysfunctions caused by menopause.

## 1 Introduction

Recent research has highlighted the critical role of estrogen homeostasis in bladder function. For instance, studies have identified significant gender disparities in the incidence of lower urinary tract dysfunction (LUTD). Female patients exhibit notably higher prevalence rates of interstitial cystitis/bladder pain syndrome (IC/BPS) (1), recurrent urinary tract infections (rUTIs) (2), and stress urinary incontinence (SUI) (3) compared to male patients. Moreover, over half of menopausal women experience the genitourinary syndrome of menopause (GSM), which encompasses urgency, dysuria, and recurrent urinary tract infections (rUTIs) (4). Research indicates that vaginal estrogen therapy significantly prevents rUTIs in peri- and post-menopausal women (5). Furthermore, our findings suggest a potential link between the downregulation of the sphingosine-1-phosphate pathway and the mechanism of dysuria in perimenopausal women (6). However, there remains a significant knowledge gap regarding how estrogen impedes the underlying mechanisms of different bladder disorders.

In recent years, there has been a growing focus on alleviating the symptoms of GSM or LUTD resulting from estrogen deprivation. Platelet-rich plasma (PRP) (7), selective androgen receptor modulators (SARMs) (8), and even testosterone (9) have demonstrated varying degrees of efficacy. Nonetheless, there is a dearth of studies mapping the macroscopic molecular effects of estrogen deprivation on bladder tissue from an omics perspective. In the past decade, the technology of omics has become increasingly mature, and omics have shown irreplaceable value in revealing disease mechanisms (10–12). Simultaneously, we have amassed extensive expertise in omics research (13–15). We posit that leveraging omics as a bridge to establish the correlation between estrogen and bladder function holds substantial promise.

In summary, we employed metabolomics to portray the metabolic profile of estrogen-deprived bladder. In this study, estrogen deprivation was simulated using the conventional ovariectomized rat model, and subcutaneous estrogen injections were administered to counteract the estrogen reduction resulting from ovariectomy. Subsequently, non-targeted metabolomics was employed to evaluate the metabolic characteristics of the urinary bladder. A comprehensive bioinformatics analysis was under-taken to investigate the potential mechanisms by which estrogen deprivation influences urinary bladder function. This research aims to establish the foundation for future investigations into the mechanisms through which estrogen modulates bladder function and for the development of innovative clinical intervention strategies.

## 2 Methods

### 2.1 Animal model

The experimental animals for this study were procured from the Experimental Animal Center of the Peking University Health Science Center. The care and treatment of the experimental animals were conducted in accordance with the guidelines provided by the Committee for the Purpose of Control and Supervision of Experiments on Animals as well as the World Medical Association Declaration of Helsinki on Ethical Principles for Medical Research involving experimental animals. The experimental protocols were approved by the ethics committee on experimental animals (No. LA2018092).

Twenty-four 12-week-old female specific pathogen-free Sprague Dawley rats were randomly assigned to three groups: sham surgery group (sham), ovariectomized group (OVX), and ovariectomized with estrogen treatment group (OVX + E). The animals were housed under controlled conditions including temperature (22–26°C), humidity (50–60%), and a 12-hour light/12-hour dark cycle. The animals were provided with non-soy feed and access to water ad libitum. The surgical procedures were performed following one week of acclimatization. The sham group underwent a skin incision and suturing, while the OVX and OVX + E group underwent ovariectomy. Starting from the 3rd day post-surgery, vaginal exfoliated cells of the rats were monitored daily for 7 consecutive days. Fourteen days after the surgery, all rats were subcutaneously administered specific drugs between 9 am and 10 am daily. The OVX + E group rats were treated with 17 β-Estradiol (25μg·kg−1·D−1; Sigma, St. Louis, Mo, USA), dissolved in ethanol and diluted with sterile sesame oil (10 mg/0.1 mL, 0.25 mL/kg; GLBIO, Montclair, CA, USA). The other two groups received the same dose of sterile sesame oil.

### 2.2 Weighing and storage of bladder samples

After 28 days of experimentation, all rats were anesthetized by intraperitoneal injection of 1% sodium pentobarbital (80mg/kg; Sigma, St Louis, MO, USA) and sacrificed. Blood was collected from the heart, centrifuged at 4°C, and the supernatant was stored at -80°C. Cardiac perfusion with cold 0.9% saline was performed before harvesting the bladder tissues. The harvested bladder samples were rinsed with 0.9% saline on ice, weighed, and stored at -80°C.

### 2.3 Radioimmunoassay test

Serum estrogen levels were determined by radioimmunoassay using the rat E2 ELISA Kit (RE1649–48T, Bioroyee, Beijing, China) according to the instructions, with a lower limit of detection of 3 pg/ml. Samples were incubated, separated, and centrifuged for detection.

### 2.4 Hematoxylin-eosin staining

Vaginal exfoliated cells were obtained by rotating a cotton swab soaked in 0.9% saline in the rats’ vagina, which were then dried, stained with hematoxylin and eosin, dehydrated with alcohol, cleared with xylene, and finally sealed with resin.

### 2.5 Bladder sample preparation in metabolomics

Accurately weigh 20 ± 1 mg of bladder tissue on ice and mix it with methanol (500 µ L) containing 5 µg/ml 2-chloro-l-phenylalanine as the internal standard. The mixture was ground for 90 seconds with a high-throughput tissue grinder (60Hz; Tissuelyser-24, Jingxin, Shanghai, China). The samples were centrifuged at 12000rpm at 4 °C for 15 minutes. Finally, 100 µL supernatant was obtained for metabolomics analysis.

### 2.6 Metabolomics measurement

The UHPLC-Q-TOF method was utilized for metabolomics detection of bladder tissue (10). Using Agilent 1290 II UPLC-QTOF 5600 PLUS (Sciex) liquid chromatography-mass spectrometry (Agilent, Lexington, MA, USA), the chromatographic column was ACQUITY UPLC HSS T3 columns (1.8 μ m. 2.1 mm × 100 mm, Waters, Dublin, Ireland), analyzed in the electric spray ionization (ESI) mode. The conditions and settings for the liquid chromatography-mass spectrometry analysis were as follows: curtain gas = 35, ion spray voltage = 5500 V (positive ion mode), ion spray voltage = -4500V (negative ion mode), temperature = 450°C, ion source gas 1 = 50, ion source gas 2 = 50.

### 2.7 Metabolomics data processing

The raw data was processed using Agilent MassHunter workstation software (version B.01.04; Agilent, Lexington, MA, USA). Isotope interference was eliminated, and the intensity threshold was set to 300 to eliminate noise. Metabolite identification was conducted by comparing with the METLIN open-source database (https://metlin.scripps.edu/landing_page.php?pgcontent=mainPage, access date: 10 October 2021).

### 2.8 Bioinformatics analysis

**Figure 1:**
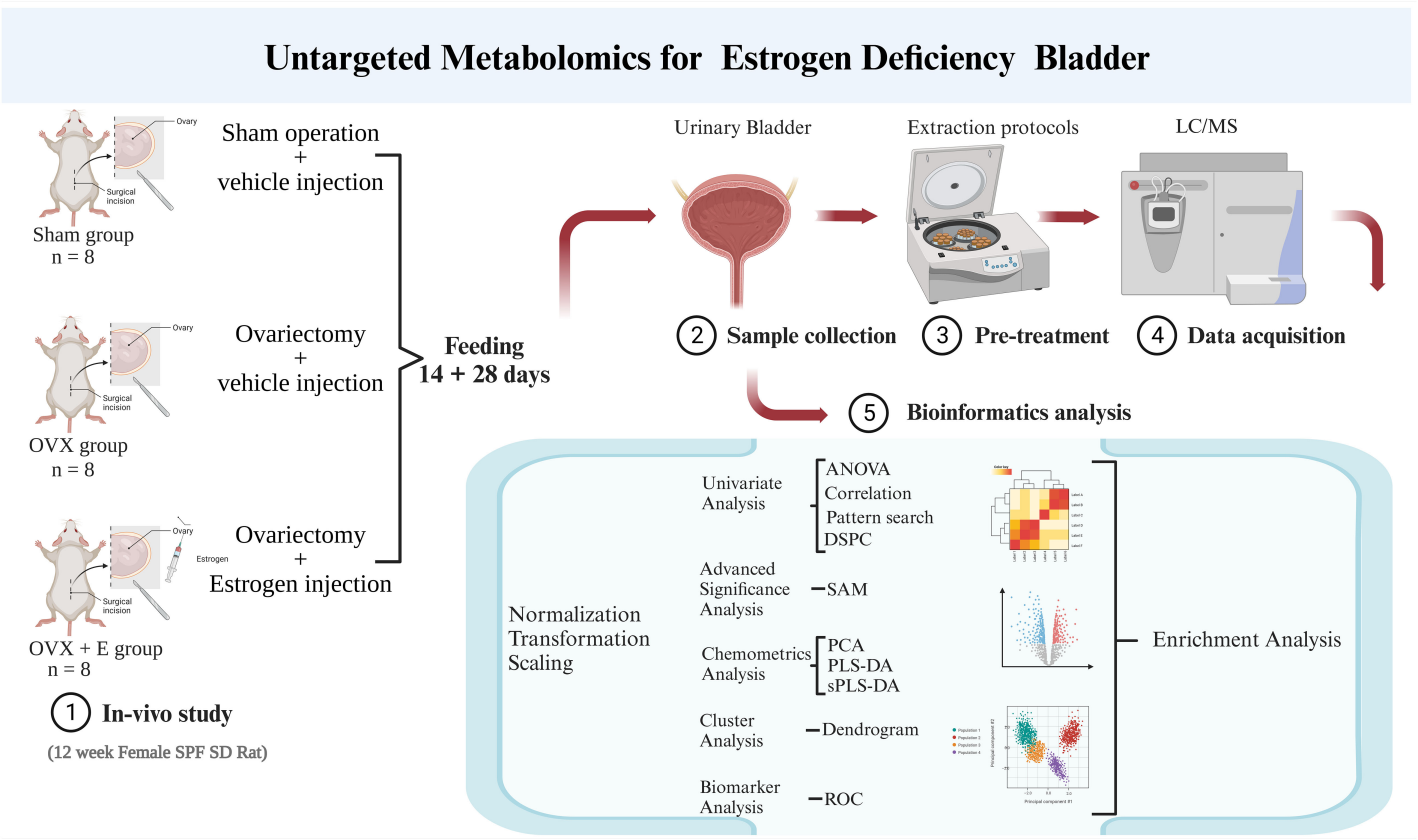
Research flowchart.

MetaboAnalyst 5.0 (http://www.metaboanalyst.ca/; Visited on October 12, 2021) was utilized for data preprocessing and bioinformatics analysis in this study (16). The group probability quotient normalization method was employed to normalize the data (17), with the sham group being the reference group. Log transformation (base 10) and the Pareto method (mean-centered and divided by the square root of the standard deviation of each variable) were used for data normalization. Various analytical methods, including Debiased Sparse Partial Correlation (DSPC) network (18), were applied for information mining. The above research process is summarized in **Figure 1**.

## 3 Results

### 3.1 Establishment of estrogen deprivation animal models

**Figure 2:**
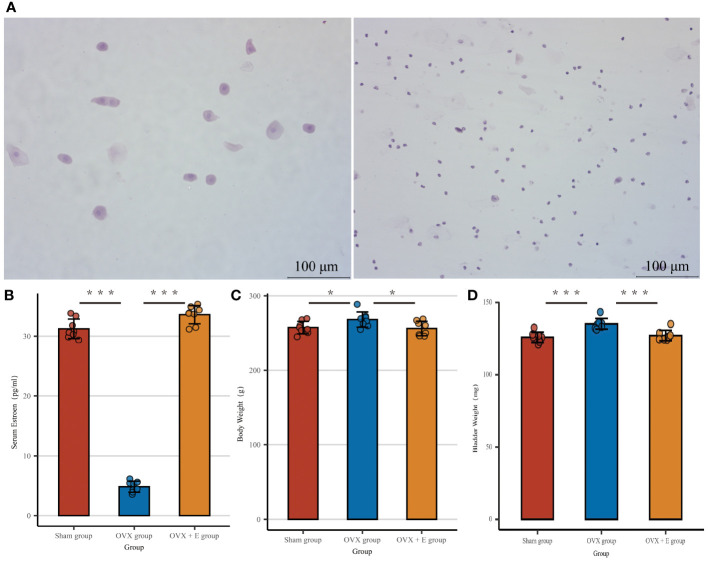
Establishment of estrogen deprivation animal Models. **(A)** The morphological characteristics of vaginal exfoliated cells, the left image shows the sham group and OVX + E group, the right image shows the OVX group. **(B)** Serum estrogen concentration, n = 8 per group. **(C)** Body weight, n = 8 per group. **(D)** Wet weight of bladder, n = 8 per group. Statistical analysis was performed using one-way ANOVA (Tukey honestly significant difference *post hoc* test) *p < 0.05, ***p < 0.001.

In order to simulate the state of estrogen deprivation, we constructed an ovariectomy rat model. The OVX group of rats that underwent ovariectomy showed significant differences in serum estrogen, vaginal exfoliated cells, bladder weight, and body weight compared to the other two groups. The cyclic morphological characteristics of vaginal exfoliated cells in the Sham group and OVX + E group rats disappeared in the OVX group rats (**Figure 2A**). At the same time, the serum estrogen level of the OVX group rats was significantly lower than the other two groups (p < 0.001, **Figure 2B**), and the body weight of the OVX group rats increased significantly compared to the other two groups (p < 0.05, **Figure 2C**), meanwhile the bladder weight increased significantly compared to the other two groups (p < 0.001, **Figure 2D**).

### 3.2 Characteristics of bladder metabolites associated with estrogen deprivation

**Figure 3:**
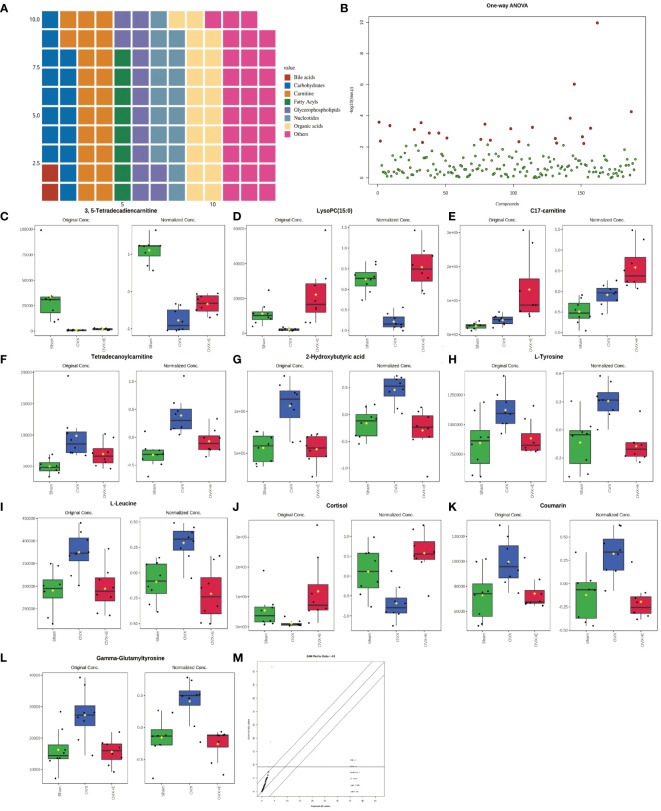
Overview of bladder metabolomics related to estrogen deprivation. **(A)** Metabolite classification statistics waffle pie chart. **(B)** Volcano diagram of differential metabolites. **(C-L)** Bar chart of the expression levels of the top ten differential metabolites calculated using the ANOVA method. **(M)** SAM analysis of differential metabolites.

Metabolomics detected 190 different metabolites in total from three groups of bladder tissues (**Supplementary Table S1**), mainly divided into eight major categories: amino acids, bile acids, carbohydrates, carnitine, fatty acyls, glycerophospholipids, nucleotides, organic acids, and others, which basically cover common bladder metabolites. Among them, amino acids account for close to 1/3 (**Figure 3A**). The metabolites and samples were subjected to calibration to approximately meet the normal distribution (**Supplementary Figures S1A, B**) for the analysis of the calibrated data.

### 3.3 Overall analysis of bladder differential metabolites related to estrogen deprivation

**Figure 4:**
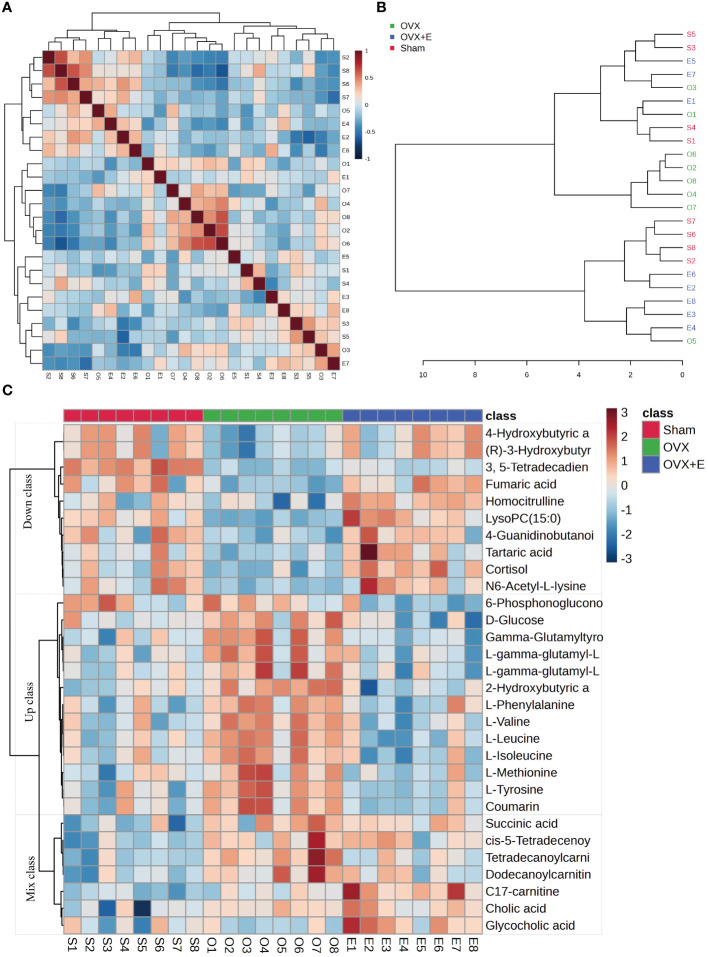
Correlation analysis of samples and differential metabolites. **(A)** Pearson correlation analysis heatmap of samples. **(B)** Hierarchical clustering tree diagram of samples. **(C)** Hierarchical clustering heatmap of samples and metabolites.

**Figure 5:**
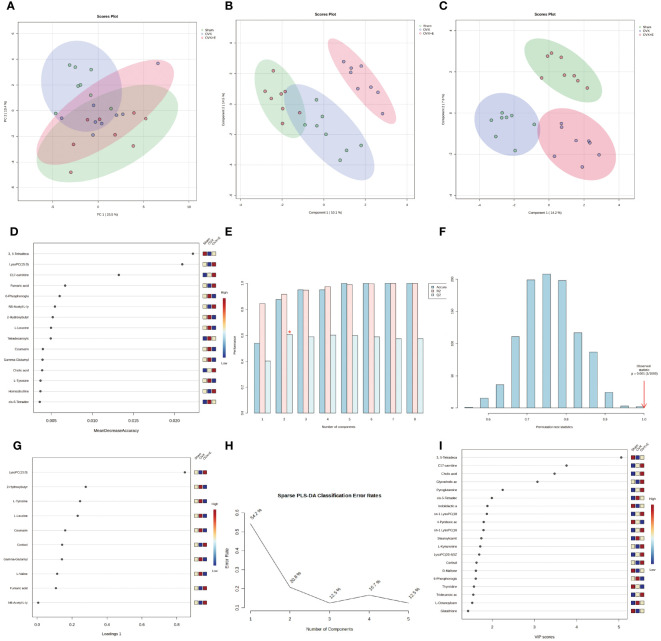
Overview of Dimension Reduction Analysis. **(A)** 2D PCA scatter plot. **(B)** 2D PLS-DA scatter plot. **(C)** 2D s- PLS-DA scatter plot. **(D)** VIP score of PLS-DA model. **(E)** 5-fold CV detection of PLS-DA model. **(F)** Permutation test of PLS-DA model. **(G)** Loading Figure of sPLS-DA Model. **(H)** Classification Error Rate of sPLS-DA Model. **(I)** VIP score of sPLS-DA model.

Using 0.05 as the false discovery rate (FDR) cutoff value, differences in metabolites among the three groups caused by estrogen deprivation were identified through one-way analysis of variance (ANOVA) (with Tukey’s Honest Significant Difference (HSD) and Fisher’s Least Significant Difference (LSD) tests as *post hoc* tests). A total of 24 differential metabolites were identified, ranked by FDR, the top 10 being: 3, 5-tetradecadiencarnitine, lysoPC (15:0), C17-carnitine, tetradecanoylcarnitine, 2-hydroxybutyric acid, l-tyrosine, l-leucine, cortisol, coumarin, and gam-ma-glutamyltyrosine (**Figures 3B-L**, **Supplementary Table S2**). The estrogen treatment did not show a restorative effect on the changes in C17-carnitine caused by ovariectomy, so it can be excluded. Further multi-class Significance Analysis of Metabolomics (SAM) was conducted based on the above results (with Delta value (FDR control) set at 4.8, **Supplementary Figure S2A**), the results indicated that 3, 5-tetradecadiencarnitine, lysoPC (15:0), C17-carnitine, and cortisol are the four most important differential metabolites (**Figure 3M**, **Supplementary Table S3**). Different metabolites have different correlations, which help to assess interference between metabolic pathways (**Supplementary Figure S2B**, **Supplementary Table S4**). Pearson correlation analysis of the three groups of samples showed that the OVX group is easier to distinguish from the Sham group and the OVX + E group, while it is more difficult to distinguish between the latter two groups, suggesting that the effect of ovariectomy can be reversed by estrogen supplementation (**Figure 4A**, **Supplementary Table S5**). The Hierarchical Clustering Dendrogram displayed the same trend (**Figure 4B**), while the Hierarchical Clustering heatmap based on the top 30 differential metabolites selected by ANOVA demonstrated three different patterns of differential expression between the metabolic group, with the Down class showing downregulation in the OVX group and upregulation in the other two groups, the Up class showing upregulation in the OVX group and downregulation in the other two groups, and the Mix class showing atypical inter-group differences (**Figure 4C**). Principal Component Analysis (PCA), Partial Least Squares Discriminant Analysis (PLS-DA), and sparse PLS-DA (sPLS-DA) were employed to conduct dimensionality reduction analysis on the metabolomic data. The unsupervised method (PCA) results indicated that principal component I accounted for 25.5% and principal component II for 13.4% (**Supplementary Figure S3A**), making it difficult to distinguish between the three groups of samples (**Figure 5A**). The other two supervised methods provided valuable evidence for differential metabolites. In the case of PLS-DA, principal component I accounted for 10.1% and principal component II for 14.5%, as depicted in **Supplementary Figure S3B**. These results illustrate significant differentiation among the three sample groups, particularly highlighting the distinctiveness of the OVX + E group in comparison to the other two, as shown in **Figure 5B**. **Figure 5C** shows the classification performance of sPLS-DA. Using a PLS-DA VIP score threshold of 2, five highly significant differential metabolites were identified: 3, 5-tetradecadiencarnitine, C17-carnitine, cholic acid, glycocholic acid, and pyroglutamine (**Figure 5D**, **Supplementary Table S6**). The 5-fold Cross validation (CV) of the PLS-DA model yielded R^2^ of 0.92, Q^2^ of 0.61, and Accuracy of 0.88 (**Supplementary Table S7**, **Figure 5E**), indicating the strong predictive performance of the PLS-DA model. Additionally, permutation test results con-firmed the model’s robustness (**Figure 5F**). Similarly, the sPLS-DA exhibited superior discrimination among the three sample groups compared to PLS-DA, with principal component I contributing 14.2% and principal component II 7.6% (**Supplementary Figure S3C**). Notably, the loading figure highlighted lysoPC (15:0) as the most significant differential metabolite, outperforming others (**Figure 5G**), and the 5-fold CV results demonstrated the optimal performance achieved by two components for the final sPLS-DA model (**Figure 5H**). Moreover, feature extraction using the random forest model resulted in an out-of-bag (OOB) error of 0.17 for the three sample groups (**Supplementary Figure S3D**). Assessing the contribution of features to classification accuracy (Mean Decrease accuracy) identified 3, 5-tetradecadiencarnitine, lysoPC (15:0), and C17-carnitine as the top three features (**Figure 5I**, **Supplementary Table S8**).

### 3.4 Subgroup analysis of diverse metabolites

**Figure 6:**
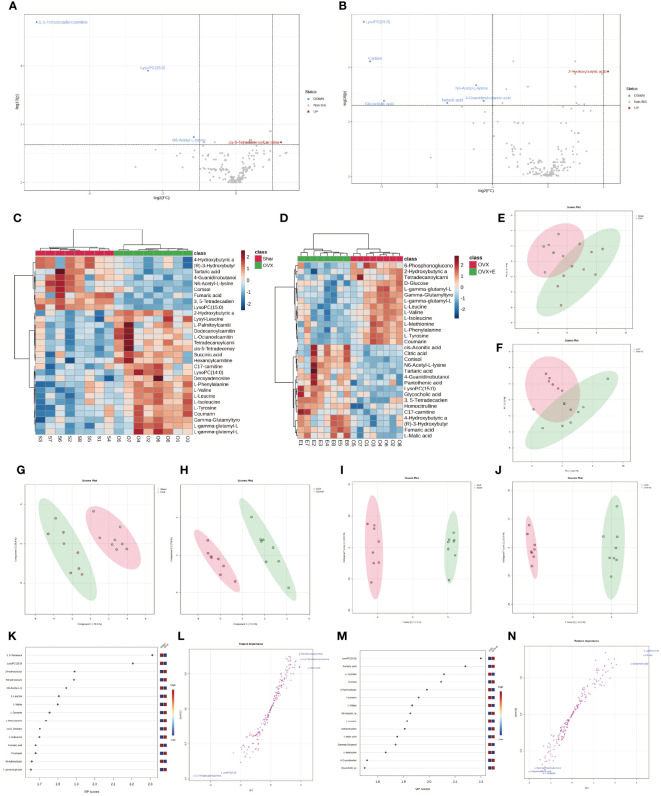
Overview of subgroup dimensionality reduction analysis. **(A)** Volcano map of differential metabolites between the sham group and OVX group. **(B)** Volcano map of differential metabolites between the OVX group and OVX + E group. **(C)** Hierarchical clustering heatmap based on TOP30 differential metabolites between sham group and OVX group. **(D)** Hierarchical clustering heatmap based on TOP30 differential metabolites between OVX group and OVX + E group. **(E)** Scatter plot of PCA between the sham group and OVX group. **(F)** Scatter plot of PCA between the OVX group and OVX + E group. **(G, H)** PLS-DA scatter plot for subgroup comparison. **(I, J)** OPLS-DA scatter plot for subgroup comparison. **(K-N)** VIP scores and S-plot plot for subgroup analysis of OPLS-DA model.

**Figure 7:**
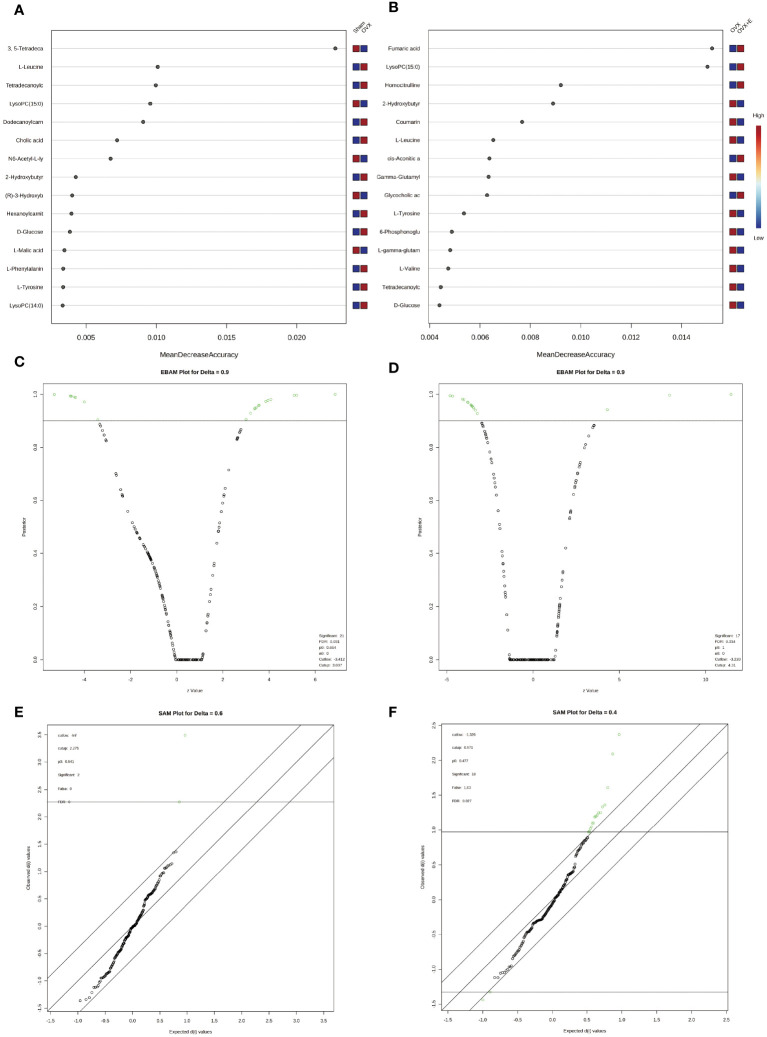
Screening differential metabolites using RF, EBAM, and SAM methods. **(A)** VIP patterns based on RF model between the sham group and OVX group. **(B)** VIP patterns based on RF model between the OVX group and OVX + E group. **(C, D)** Volcano plots for subgroup comparison based on EBAM method. **(E, F)** Screening of differential metabolites between subgroups based on SAM method.

In order to accurately screen and reduce the dimensions of the bladder metabolism characteristics associated with estrogen deprivation identified in our multi-component analysis, we comprehensively used the methods of t-test, PCA, PLS-DA and Orthogonal PLS-DA (OPLS-DA), Empirical Bayesian Analysis of Metabolomics (EBAM), and Random Forest to perform inter-group analysis between the OVX group and the sham group, as well as between the OVX group and the OVX + E group. The distribution be-fore and after data correction is presented in **Supplementary Figure S4**. With a Fold Change (FC) threshold set at 2 and an FDR set at 0.05, inter-group t-tests for metabolites were con-ducted. Compared to the sham group, the up-regulated metabolites in the OVX group were cis-5-tetradecenoylcarnitine, and the down-regulated metabolites were N6-acetyl-L-lysine, lysoPC (15:0), and 3,5-tetradecadiencarnitine. In comparison to the OVX + E group, the up-regulated metabolite in the OVX group was 2-hydroxybutyric acid, while the down-regulated metabolites were 4-guanidinobutanoic acid, N6-acetyl-l-lysine, tartaric acid, glycocholic acid, cortisol, and lysoPC (15:0) (**Figures 6A, B**, **Supplementary Table S9**). The hierarchical clustering heat map based on the top 30 different metabolites clearly showed the expression trends of different metabolites among different group (**Figures 6C, D**). Three methods, PCA, PLS-DA, and OPLS-DA, were employed to perform dimensionality reduction analysis on inter-group data (**Figures 6E-N**). Firstly, the unsupervised method (PCA) maintained a certain degree of differentiation between the Sham and OVX group (**Figure 6E**). Principal component I reached 30.1%, principal component II reached 17.4%, accumulating to 47.5% (**Supplementary Figures S5A, B**), but the differentiation between the OVX and OVX + E group was insufficient (**Figure 6F**), with principal component I at 26.2% and principal component II at 15%, accumulating to 41.2% (**Supplementary Figures S5C, D**). The supervised methods (PLS-DA and OPLS-DA) demonstrated excellent intergroup differentiation (**Figures 6G-J**), but the permutation test results indicated over-fitting of the PLS-DA model (**Supplementary Figures S5E, F**). The cross-validation results indicated that the R^2^X for the OPLS-DA model of OVX versus sham group was 0.17, R^2^Y was 0.79, and Q^2^ was 0.58 (**Supplementary Figure S5G**). The permutation test yielded an R^2^Y of 0.99 (p = 0.007) and Q^2^ of 0.68 (p = 0.002) (**Supplementary Figure S5H**), indicating a good fit for the OPLS-DA model. Metabolites with VIP scores exceeding 2 (lysoPC (15:0) and 3,5-tetradecadiencarnitine) also demonstrated significant importance in the S-plot (**Figures 6K, L**, **Supplementary Table S10**). The cross-validation results indicated that the R^2^X for the OPLS-DA model of OVX versus OVX + E group was 0.15, R^2^Y was 0.87, and Q^2^ was 0.64 (**Supplementary Figures S5I**). The permutation test yielded an R^2^Y of 0.99 (p < 0.001) and Q^2^ of 0.86 (p < 0.001) (**Supplementary Figure S5J**), confirming a perfect fit for the OPLS-DA model. Among the metabolites with VIP scores exceeding 2 (lysoPC (15:0), fumaric acid, l-tyrosine, and cortisol), except for fumaric acid, the other three also showed significant importance in the S-plot (**Figures 6M, N**, **Supplementary Table S11**). Additionally, from the perspective of machine learning, we carried out supplementary screening of potential different metabolites using a representative random forest tree(RF) model to evaluate inter-group differences. The classification error rate graph is shown in **Figures S5K, L**. The VIP plot, based on features ranked by their contributions to classification accuracy, indicated that 3,5-tetradecadiencarnitine contributed the most to the RF model between the sham and OVX group, while fumaric acid and lysoPC (15:0) contributed the most to the RF model between the OVX and OVX + E group (**Figures 7A, B**). Lastly, we applied EBAM and SAM evaluation methods (**Figures 7C-F**). EBAM results indicated that the most valuable different metabolites between the sham and OVX group were 3,5-tetradecadiencarnitine, lysoPC (15:0), tetradecanoylcarnitine, and 2-hydroxybutyric acid, while the different metabolites between the OVX and OVX + E group were lysoPC (15:0), l-tyrosine, fumaric acid, cortisol, l-valine, coumarin, and 2-hydroxybutyric acid (**Supplementary Table S12**). SAM analysis yielded results consistent with EBAM, showing that the most valuable different metabolites between the sham and OVX group were 3,5-tetradecadiencarnitine and lysoPC (15:0), while between the OVX and OVX + E group, they were lysoPC (15:0), cortisol, and glycocholic acid (**Supplementary Table S13**).

### 3.5 Exploration of metabolite expression patterns

**Figure 8:**
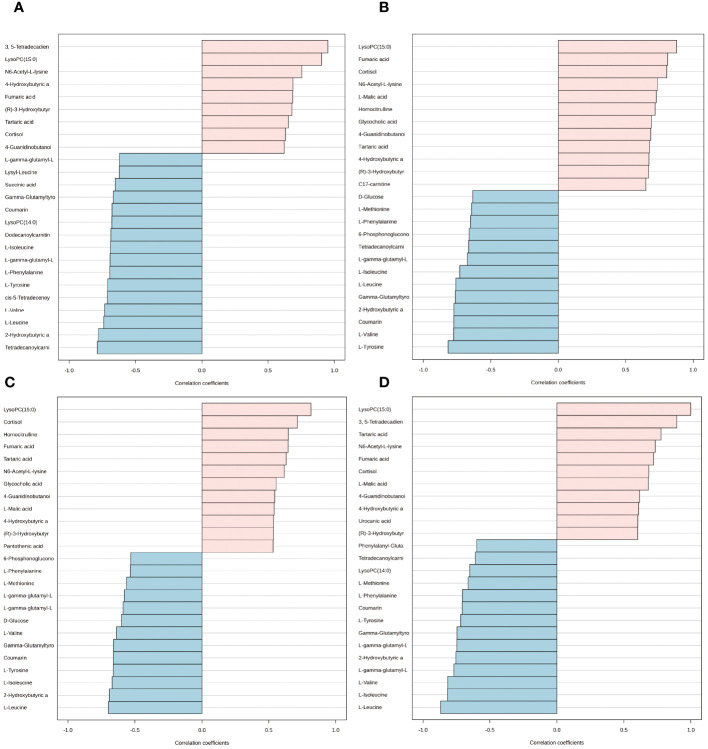
Expression patterns of metabolites. **(A)** Metabolite expression patterns associated with ovariectomy. **(B)** Metabolite expression patterns related to estrogen supplementation. **(C)** Metabolite expression patterns associated with increased estrogen concentration. **(D)** Metabolite expression patterns associated with LysoPC (15:0).

We employed the pattern search method to explore the metabolite expression patterns related to estrogen deprivation. The metabolite expression pattern associated with OVX is shown in **Figure 8A**. The positive correlation coefficients of 3, 5-tetradecadiencarnitine with lysoPC (15:0) exceed 0.9, while the negative correlation coefficient of tetradecanoylcarnitine with 2-hydroxybutyric acid also exceeds -0.77 (**Supplementary Table S14**). The metabolite expression pattern related to estrogen supplementation is shown in **Figure 8B**, where the positive correlation coefficients of lysoPC (15:0), fumaric acid, and cortisol exceed 0.8, and the negative correlation coefficients of l-tyrosine, l-valine, coumarin, and 2-hydroxybutyric acid also exceed -0.77 (**Supplementary Table S15**). Lastly, to obtain the metabolite expression pattern most relevant to estrogen, in the order of estrogen content from low to high, three groups were studied—OVX group, sham group, and OVX + E group. The metabolites most significantly positively correlated with this pattern were lysoPC (15:0) and cortisol, while the metabolites most significantly negatively correlated with this pattern were l-leucine and 2-hydroxybutyric acid (**Figure 8C**, **Supplementary Table S16**). Given the close association between lysoPC (15:0) and estrogen, we further explored the metabolite expression pattern related to lysoPC (15:0), and the results show that the strongest correlation is between 3, 5-tetradecadiencarnitine and lysoPC (15:0), with a correlation coefficient of 0.89. Additionally, the expression pattern of three branched-chain amino acids, l-leucine, l-isoleucine, and l-valine, exhibited negative correlation coefficients with lysoPC (15:0) reaching -0.8. Furthermore, the expression pattern of lysoPC (14:0) is negatively correlated with that of lysoPC (15:0) (**Figure 8D**, **Supplementary Table S17**).

### 3.6 Validation of typical biomarkers

**Figure 9:**
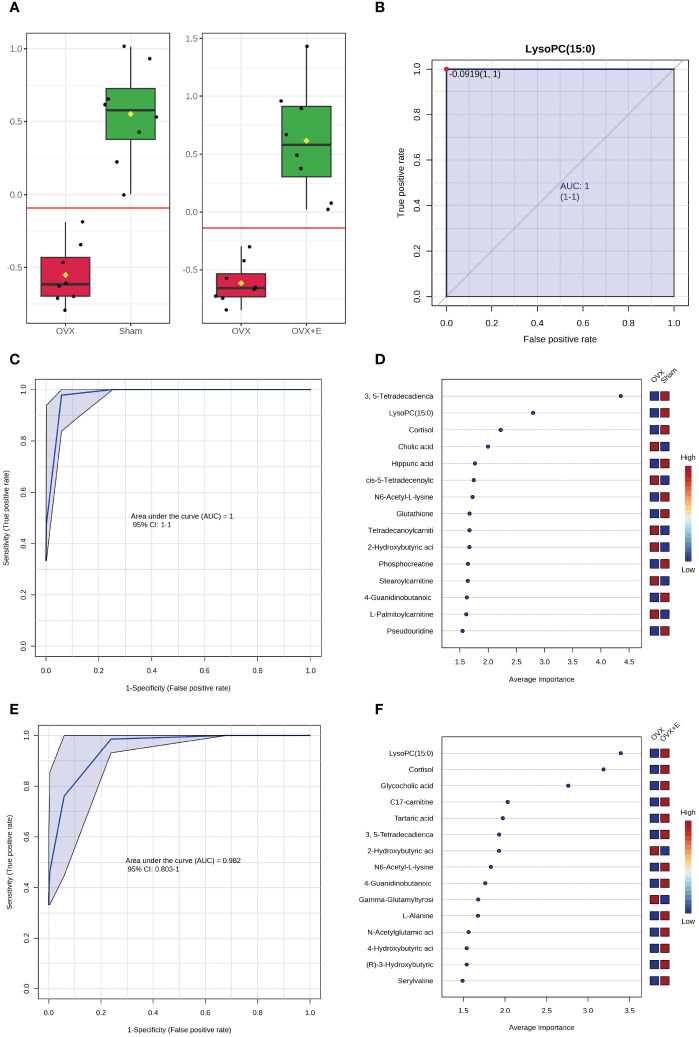
Screening of typical biomarkers. **(A)** Content bar charts of LysoPC (15:0) in different subgroups. **(B)** ROC curve of LysoPC (15:0). **(C)** Multivariate ROC curve based exploratory analysis between the sham and OVX group. **(D)** Average importance ranking of multivariate models for biomarkers between the sham and OVX group. **(E)** Multivariate ROC curve based exploratory analysis between the OVX and OVX + E group. **(F)** Average importance ranking of multivariate models for biomarkers between the OVX and OVX + E group.

The application of classical univariate ROC curve analysis entails the generation of ROC curves, calculation of AUC or partial AUC with their corresponding 95% confidence intervals, identification of optimal cutoffs for specific features, and the development of performance tables for sensitivity, specificity, and confidence intervals at different cutoff points. Notably, whether comparing the sham and OVX group or the OVX and OVX + E group, the AUC value for lysoPC (15:0) is consistently 1 (**Figures 9A, B**, **Supplementary Table S18**). Furthermore, the multivariate ROC curve-based exploratory analysis (based on PLS-DA) demonstrates highly effective diagnostic performance in distinguishing between the sham and OVX group, yielding an AUC of 1 (**Figure 9C**). The misclassification plot highlights a complete differentiation between the sham and OVX group, with no misclassified samples (**Supplementary Figure S6A**). Additionally, the ROC prediction accuracy for models constructed with five or more features consistently maintains a 100% success rate (**Supplementary Figure S6B**). The top three features of highest average importance in this multi-variate model are 3,5-tetradecadiencarnitine, lysoPC (15:0), and cortisol (**Figure 9D**, **Supplementary Table S19**). However, the diagnostic efficiency of this method slightly decreases when comparing the OVX and OVX+E group, with an AUC value of 0.98 (**Figure 9E**). The misclassification plot illustrates that a sample denoted as O-5 from the OVX group has been misclassified as belonging to the sham group (**Supplementary Figure S6C**). Notably, the ROC prediction accuracy for models built with five features is 89.7%, and with an increase in the number of features to 50, the maximum prediction accuracy reaches 96.7% (**Supplementary Figure S6D**). The top three features of highest average importance in this multivariate model are lysoPC (15:0), cortisol, and glycocholic acid (**Figure 9F**, **Supplementary Table S20**). In summary, lysoPC (15:0) and cortisol serve as relatively effective diagnostic biomarkers.

### 3.7 Comprehensive analysis of differential metabolites

**Figure 10:**
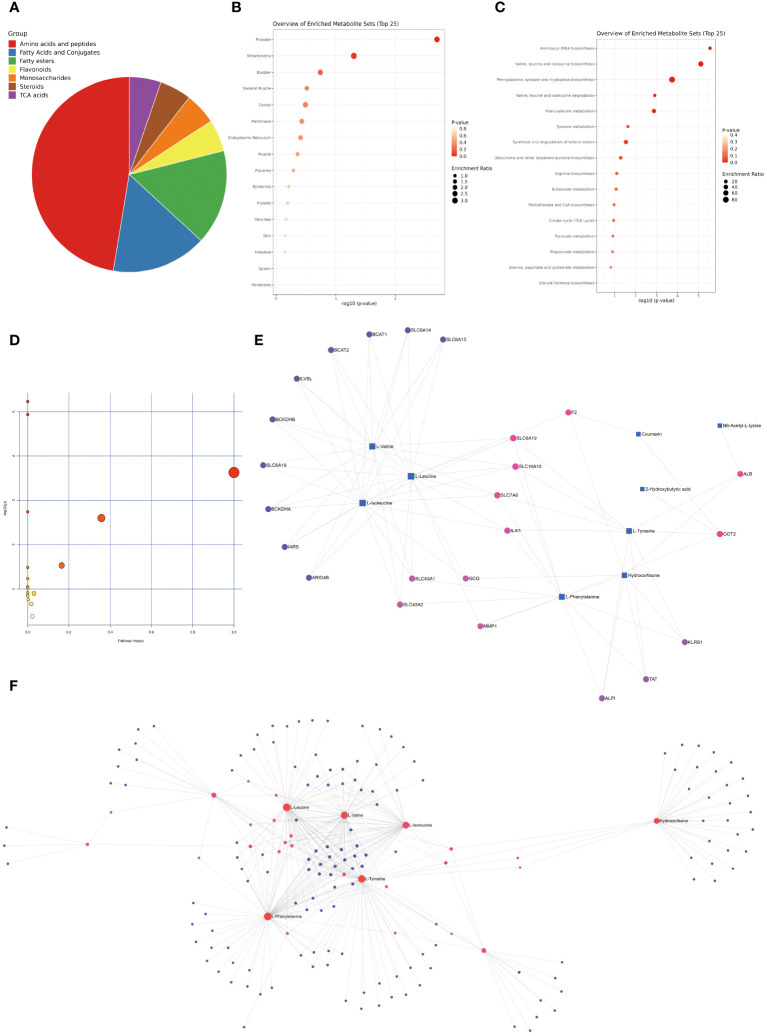
Comprehensive Analysis of Differential Metabolites. **(A)** Classification pie chart of differential metabolites. **(B)** Organ enrichment analysis of differential metabolites. **(C)** KEGG enrichment analysis of differential metabolites. **(D)** Enrichment analysis of rat specific metabolite pathways. **(E)** Gene-Metabolite Interaction Network based on differential metabolites. **(F)** Metabolite-Metabolite Interaction Network based on differential metabolites.

The selected differential metabolites were comprehensively analyzed in combination with the above analysis results. From a classification perspective, the proportion of amino acids and peptides exceeded 50%, while the proportions of fatty acids and conjugates, as well as fatty esters, exceeded one quarter. The remaining major categories included flavonoids, monosaccharides, TCA acids, and steroids (**Figure 10A**). Regarding the organ distribution of differential metabolites, the prostate, mitochondria, and bladder-specific metabolites occupied the top three positions (**Figure 10B**). The KEGG enrichment analysis results indicated that the most significant five pathways included aminoacyl-tRNA biosynthesis, valine leucine and isoleucine biosynthesis, phenylalanine tyrosine and tryptophan biosynthesis, valine leucine and isoleucine degradation, and phenylalanine metabolism (**Figure 10C**, **Supplementary Table S21**). We summarized the rat-specific metabolic pathways, and the results indicated that the most meaningful metabolic pathways were phenylalanine, tyrosine, and tryptophan biosynthesis, and phenylalanine metabolism (**Figure 10D**, **Supplementary Table S22**). Lastly, based on the differential metabolites, the Gene-Metabolite Interaction Network explored the interactions between function-ally related metabolites and genes, showing a close association between SLC gene family and differential metabolites (**Figure 10E**). The Metabolite-Metabolite Interaction Network results indicated potential functional relationships among a series of differential metabolites, including three branched-chain amino acids (L-leucine, L-valine, L-isoleucine), two aromatic amino acids (L-phenylalanine, L-tyrosine), and hydrocortisone (**Figure 10F**).

## 4 Discussion

The modeling effect of the ovariectomy rat model is stable, and this model has irreplaceable value for studying the effect of estrogen deprivation. The increase in body weight in the ovariectomized rat is consistent with previous studies, at the mechanistic level, estrogen’s inhibitory effect on the increase in body weight in the ovariectomy model may be related to the regulation of physical activity, fat distribution, energy expenditure, and leptin sensitivity (19, 20). Differences have been observed in the changes in bladder weight in previous models of ovariectomized rats and rabbits, which may be related to varying proportions of muscle atrophy and collagen fiber replacement (21–24). Although this study did not deeply investigate the bladder function, the susceptibility to OAB and UTI caused by the ovariectomy animal model has been widely confirmed (7, 25–27). It is well-known that estrogen receptors are widely distributed in the bladder (28), and obviously, estrogen can to some extent regulate bladder function. The bladder metabolic characteristics related to estrogen deprivation depicted by the ovariectomy model in female rats reflects the overall metabolic trend of the bladder. Based on the above findings, it is certainly a challenging task to depict the possible association between metabolites and bladder functional changes.

Our study combined various statistical analysis methods and identified a series of valuable differential metabolites. Among the numerous candidate metabolites, 3, 5-tetradecadiencarnitine, lysoPC (15:0), and cortisol are considered the most promising biomarkers. 3, 5-tetradecadiencarnitine is a long-chain acylcarnitine compound mainly located in the extracellular space and near the membranes. The primary function of most long-chain acylcarnitines is to ensure the transport of long-chain fatty acids into the mitochondria (29). The current research found that ovariectomy significantly decreased the levels of 3, 5-tetradecadiencarnitine and that this decrease could be restored by estrogen supplementation. Similarly, Guo et al. (30) found that the levels of acylcarnitines in the intestines of ovariectomized mice were significantly lower than those in the sham group, which may be related to the changes in intestinal flora induced by estrogen. Overall, there is limited research on 3, 5-tetradecadiencarnitine, and its involvement in the development of diseases has been rarely reported. However, some studies have found that 3,5-tetradecadiencarnitine is a good biomarker for various metabolic disorders such as diabetes, cardiovascular diseases, and obesity (31, 32). Further research is needed to explore whether the decrease of 3, 5-tetradecadiencarnitine in the bladder under estrogen deprivation is mediated by changes in fatty acid transport and mitochondrial energy metabolism. Although there is no unified conclusion on the effects of menopause on the levels and rhythms of cortisol (33–35), this study is the first to discover that estrogen deprivation can lead to a decrease in the cortisol concentration of bladder tissue. Recent studies have found that plasma cortisol is involved in regulating the day-night micturition rhythm of the bladder (36). However, there is still no research that can elucidate how cortisol in the bladder exerts its effects. Considering the important role of cortisol itself in substance metabolism and immune regulation (37), the correlation between bladder tissue cortisol levels and bladder dysfunction may be related to changes in immune function and substance metabolism patterns. LysoPC (15:0) is a shorthand for lysophosphatidylcholine (15:0) and is a saturated form of lysoPC. Elevated levels of lysoPC in body fluids have been identified as good biomarkers in inflammatory and immune-related diseases such as allergic asthma, rheumatoid arthritis, and osteoarthritis (38–42). LysoPC exists in numerous subtypes, each exhibiting distinct effects in various diseases. For example, lysoPC (16:0) is elevated in the bone marrow of rheumatoid arthritis patients and mediates pain symptoms in the rheumatoid arthritis animal model (43). LysoPC (18:1) and lysoPC (16:0) can induce mechanical and cold hypersensitivity, respectively (44, 45), while lysoPC (14:0) can lower blood pressure and renal blood flow in rats (46). Surprisingly, there is currently a lack of research on the correlation between lysoPC and bladder function. Exploring the mechanisms through which lysoPC (15:0) regulates bladder function in the context of estrogen deprivation is an area worthy of scientific exploration.

Regardless of the classification of differential metabolites or the results of KEGG pathway analysis, they all suggest that the imbalance of amino acid metabolism caused by estrogen deprivation may be the main mediator of bladder dysfunction. In this context, aminoacyl-tRNA is involved in amino acid biosynthesis pathways, and the amino acids of particular interest include two types: branched-chain amino acids (Valine, leucine and isoleucine) and aromatic amino acids (Phenylalanine, tyrosine and tryptophan). In the current study, the levels of branched-chain amino acids in the bladders of ovariectomized rats were higher compared to the control group, and estrogen supplementation was able to suppress these changes. Although branched-chain amino acids are often considered as good nutritional supplements, in recent decades, they have been implicated in exerting negative effects in metabolic disorders such as obesity and diabetes. For example, in obesity and diabetes, the levels of blood branched-chain amino acids show an upward trend (47, 48), and after obesity and metabolic disorders are corrected, the levels of plasma branched-chain amino acids decrease accordingly (49, 50). Controlling the intake of branched-chain amino acids helps improve metabolic disorders in rodents and prolong lifespan, while an increase in their intake exacerbates adverse outcomes associated with metabolic imbalance (51, 52). Therefore, an increase in the levels of branched-chain amino acids in populations with metabolic disorders may mediate the occurrence of adverse outcomes. The population deprived of estrogen itself faces metabolic disorders such as obesity and abnormal glucose tolerance, and the increase of branched-chain amino acids in the bladder is likely closely related to the metabolic dysfunction of the bladder. Similarly, aromatic amino acids are also associated with the burden of metabolic diseases such as diabetes and cardiovascular diseases, and in a large-scale study involving more than 26,000 participants, it was found that the concentrations of tyrosine and isoleucine in the blood samples of postmenopausal women were higher than those of premenopausal women (53). For female IC/BPS patients, the increase of phenylalanine in the urine is considered to be a good biomarker (54). The correlation between aromatic amino acids, represented by phenylalanine, and bladder dysfunction caused by estrogen deprivation, is still a topic worthy of exploration. The organ distribution of differential metabolites is mainly located in the prostate, mitochondria, and bladder. We speculate that the enrichment of prostate-specific metabolites is mainly related to the imbalance of estrogen-androgen balance caused by the decrease in estrogen, while the regulatory effect of estrogen on the structure and function of mitochondria has been found in multiple diseases (55, 56). Whether the regulatory effect of estrogen on bladder function is related to mitochondria is another research direction that deserves attention.

The current study is an exploratory study of the bladder metabolic characteristics related to estrogen deprivation. Although certain positive results have been obtained, there are still inevitable flaws. Firstly, despite the support of ample research evidence, this study did not further verify the changes in bladder function due to estrogen deprivation, such as detrusor contraction function, epithelial anti-infection ability, and aging indicators. Secondly, the positive results obtained in this study lack supporting evidence from human studies. Finally, no further phenotype and mechanism exploration have been conducted on the promising metabolites selected in this study.

## 5 Conclusions

The current research simulated the metabolic effects of estrogen deprivation on the bladder using the classic ovariectomized model. This study is the first to demonstrate the metabolic map of the bladder under estrogen deprivation, highlighting the metabolic characteristics associated with amino acid metabolic disorder. Additionally, the study identified promising biomarkers, including 3,5-tetratecadiencarnitine, lysoPC (15:0), and cortisol. Furthermore, it provides a solid theoretical foundation for precise intervention measures in bladder dysfunction resulting from menopause.

## Data availability statement

The original contributions presented in the study are included in the article/**Supplementary Material**. Further inquiries can be directed to the corresponding authors.

## Ethics statement

The animal study was approved by the animal ethics committee of Peking University Health Science Center. The study was conducted in accordance with the local legislation and institutional requirements.

## Author contributions

WZ: Writing – original draft, Visualization, Software, Resources, Funding acquisition, Formal analysis, Data curation. QY: Writing – original draft, Formal analysis. YS: Writing – original draft, Formal analysis. WL: Writing – review & editing, Project administration, Conceptualization. YL: Writing – review & editing, Supervision, Project administration, Methodology, Funding acquisition, Conceptualization.
