# Supplementary material for: Exploratory metabolomic analysis for characterizing the metabolic profile of the urinary bladder under estrogen deprivation
Source: Front Endocrinol (Lausanne). 2024 May 31;15:1384115. doi: 10.3389/fendo.2024.1384115 (PMC11176512; doi:10.3389/fendo.2024.1384115)
Supplement: Supplementary file 14 [file DataSheet_14.docx]

Supplementary Material


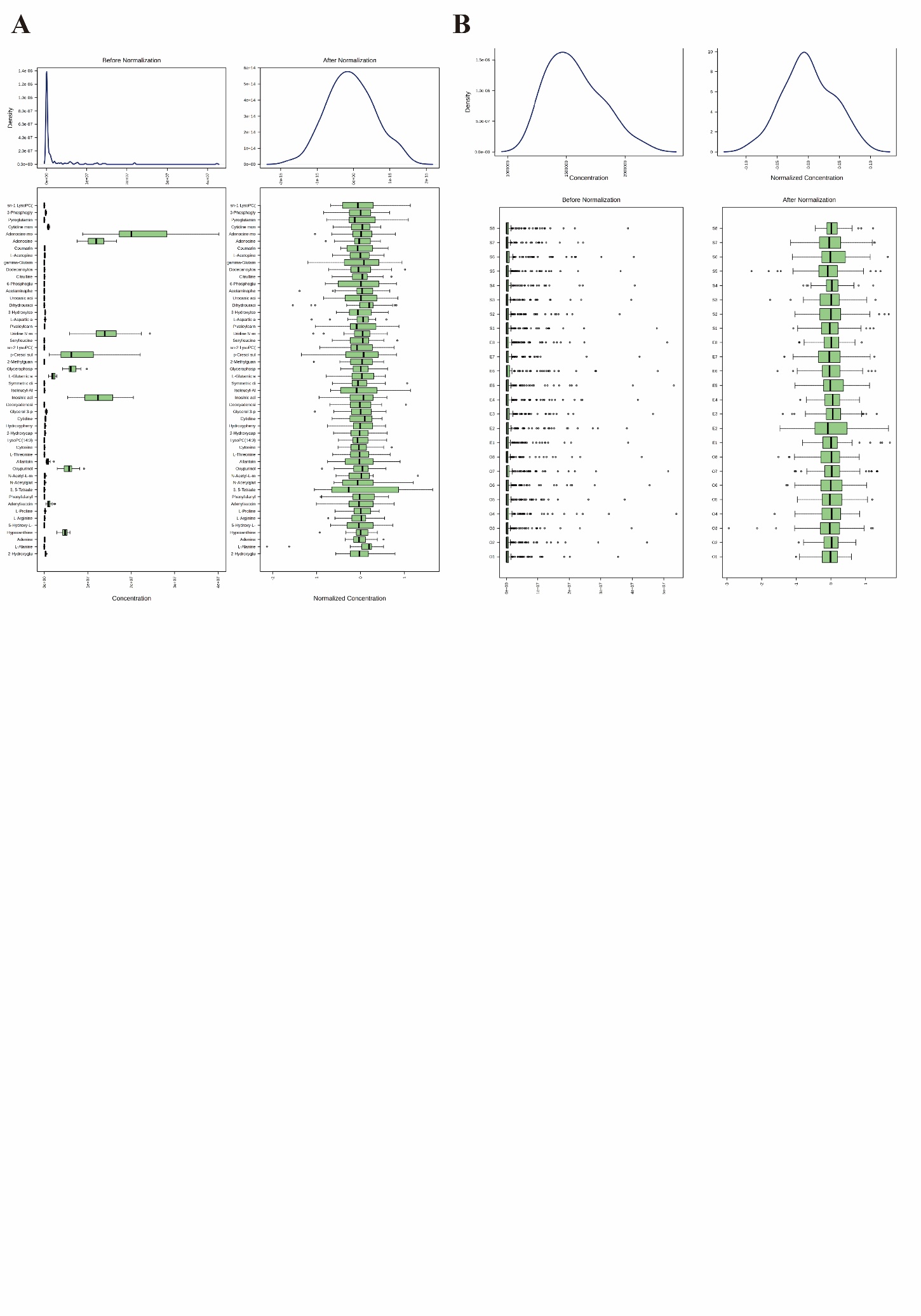


Figure S1: Normalization effect of metabolite and sample data. (A)Normalization effect of metabolites. (B) Normalization effect of samples.


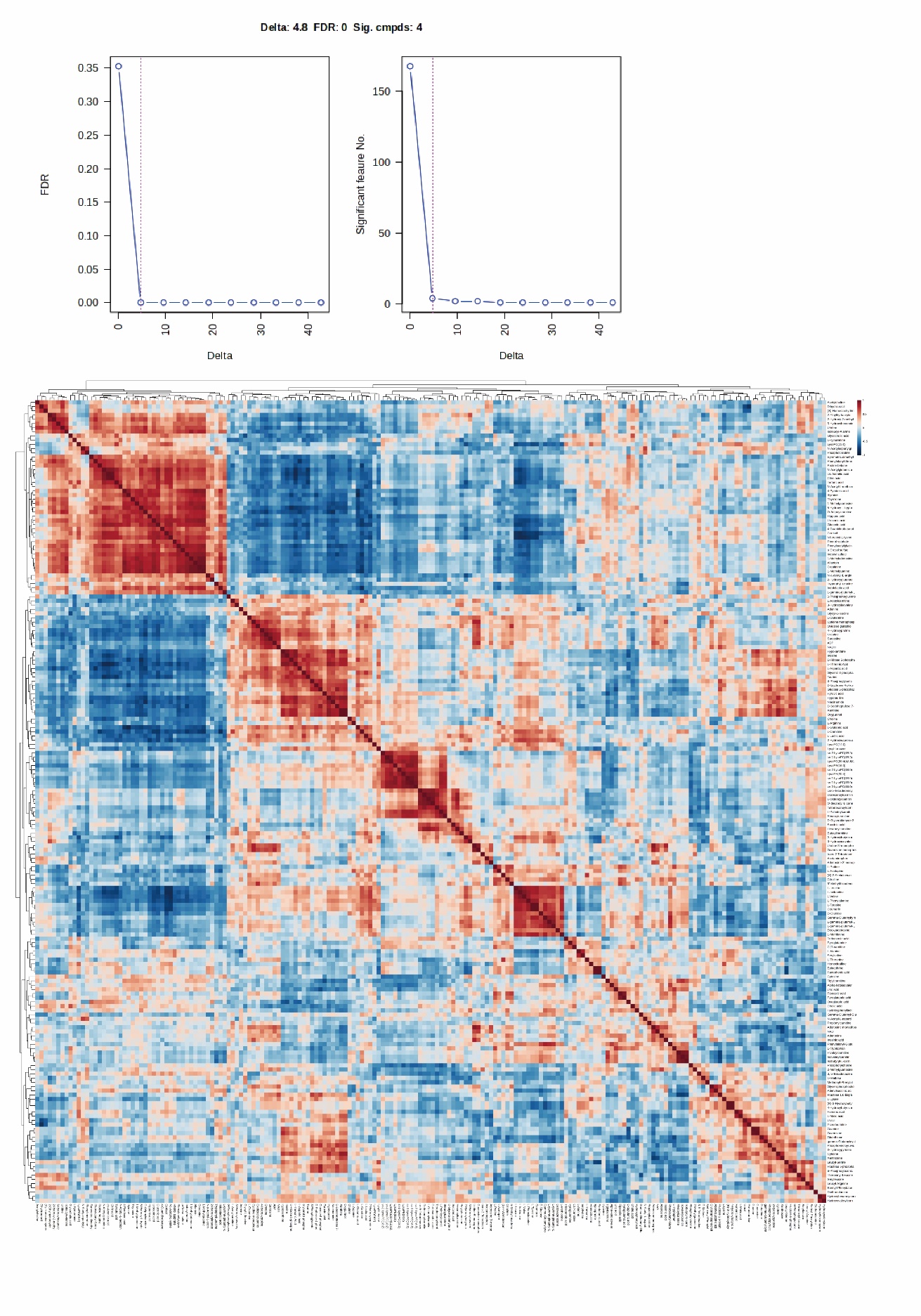


Figure S2：Importance analysis and correlation heatmap of metabolites. (A) Importance analysis of metabolites .(B) Correlation heatmap of metabolites.


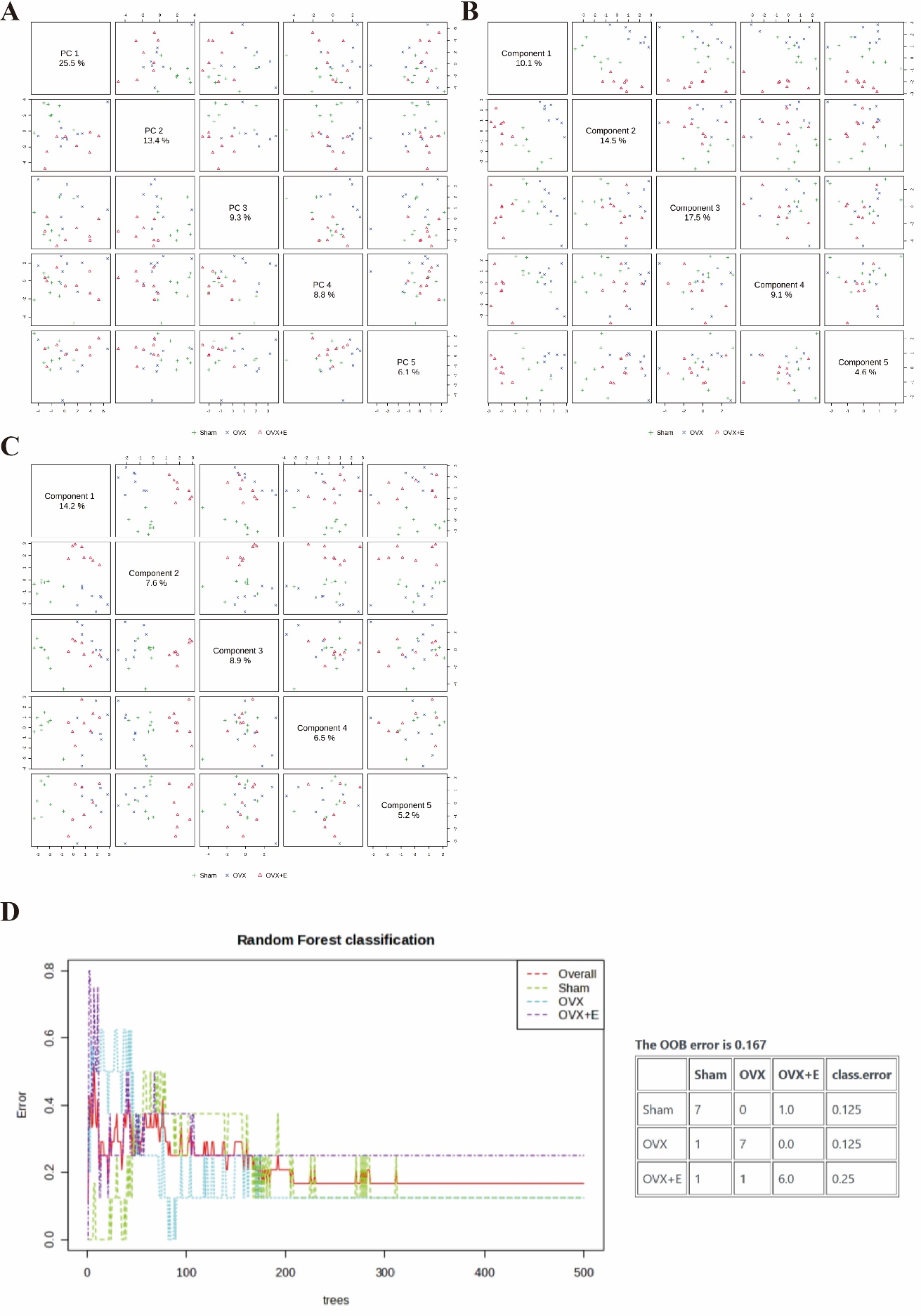


Figure S3：Dimensionality reduction analysis and random forest tree model of metabolomics data. (A) The results of PCA. (B) The results of PLS-DA. (C) The results of sPLS-DA. (D) The random forest model.


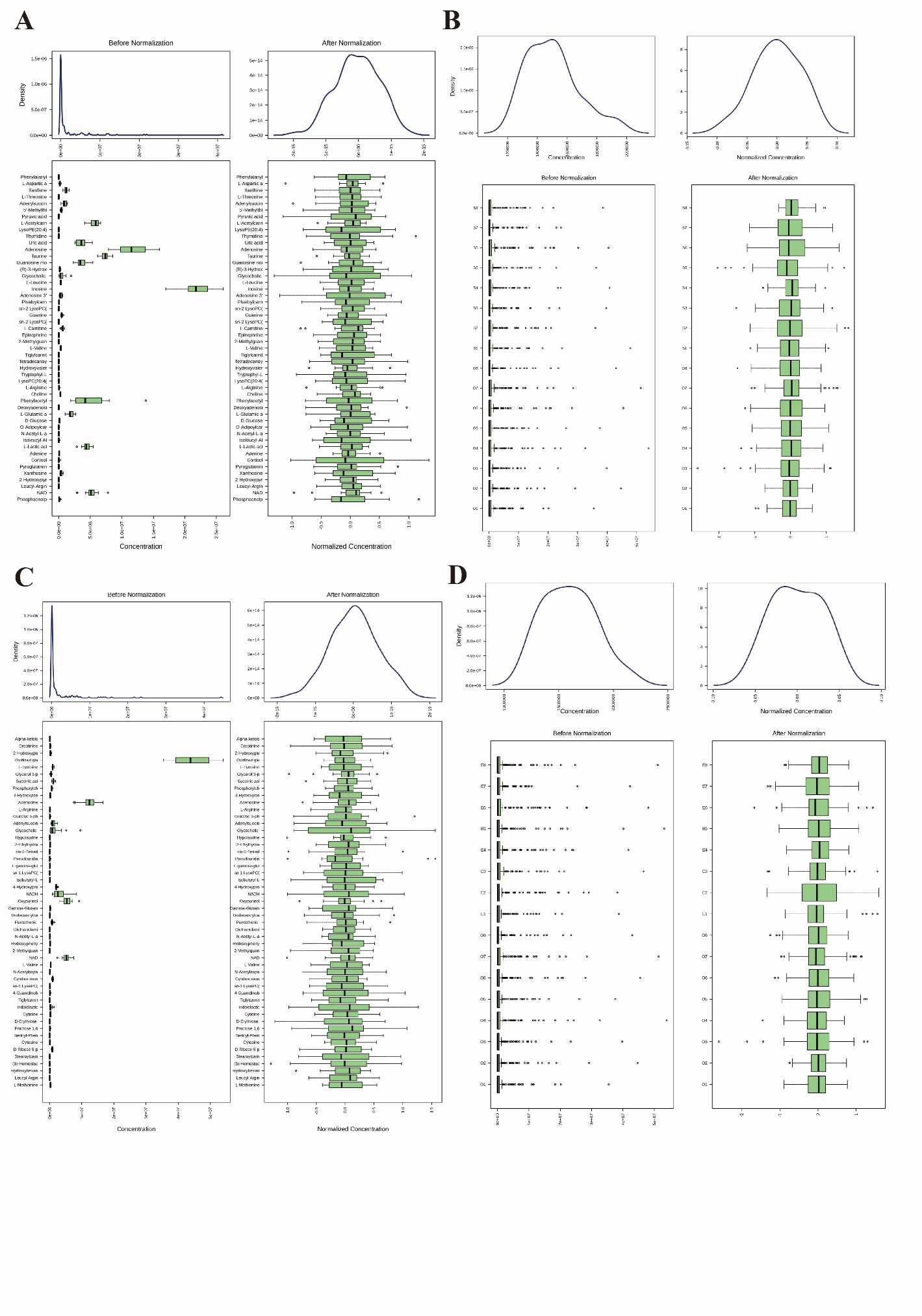


Figure S4：Normalization effect of metabolites and samples in subgroup analysis. (A)Normalization effect of metabolites between sham group and OVX group. (B) Normalization effect of samples between sham group and OVX group. (C)Normalization effect of metabolites between OVX group and OVX + E group. (D) Normalization effect of samples between OVX group and OVX + E group.


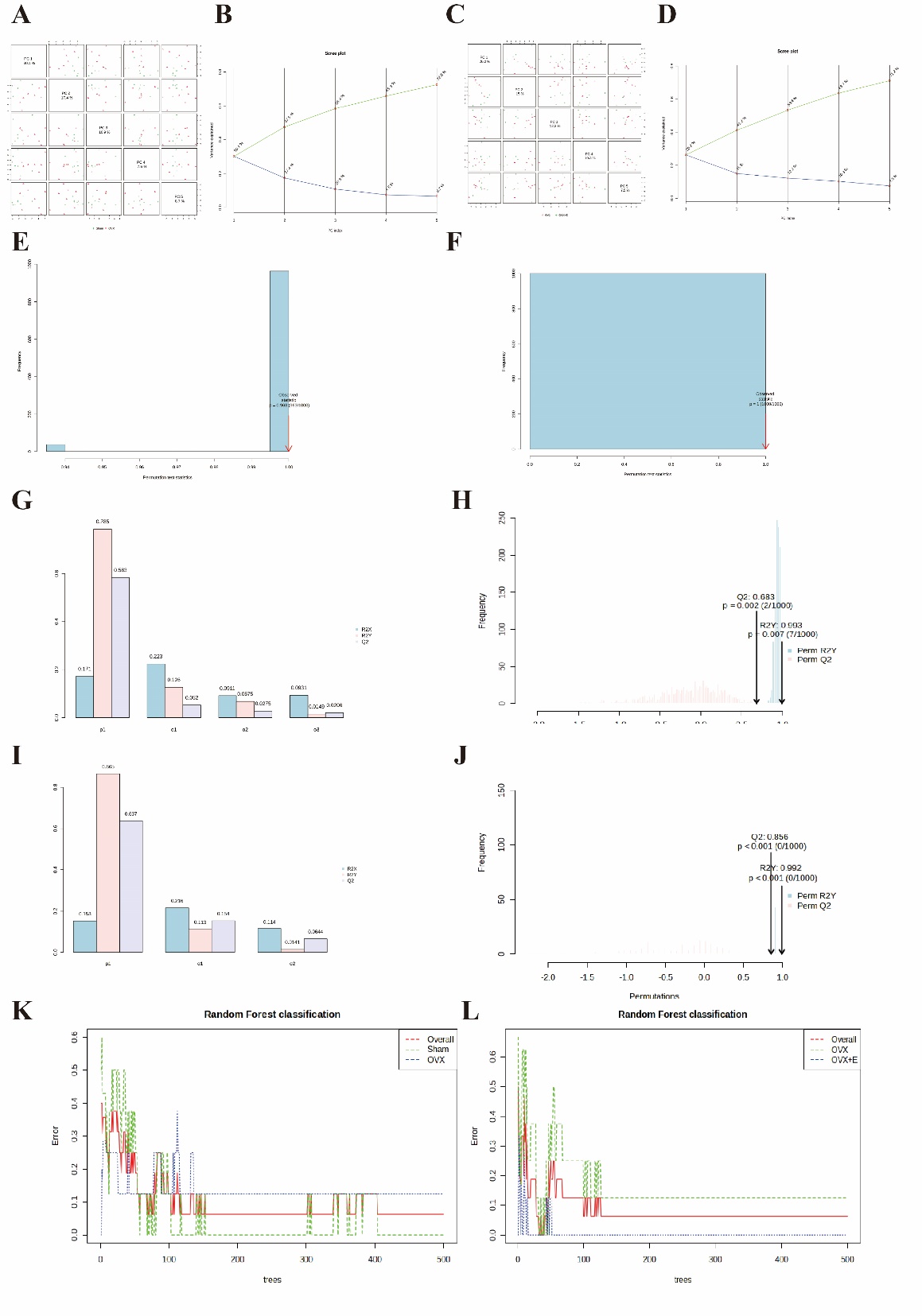
Figure S5：Dimension reduction analysis results between sub-groups. (A) PCA scatter plot between sham group and OVX group. (B) PCA scatter plot between sham group and OVX group. (C) PCA score plot between OVX group and OVX + E group. (D) PCA score plot between OVX group and OVX + E group. (E-F) permutation test of the PLS-DA model.(G) The cross-validation results for the OPLS-DA model of OVX versus sham group. (H) The permutation test for the OPLS-DA model of OVX versus sham group. (I) The cross-validation results for the OPLS-DA model of OVX versus OVX + E group. (J) The permutation test for the OPLS-DA model of OVX versus OVX + E group. (K) The classification error rate graph between sham group and OVX group. (L) The classification error rate graph between OVX group and OVX + E group.


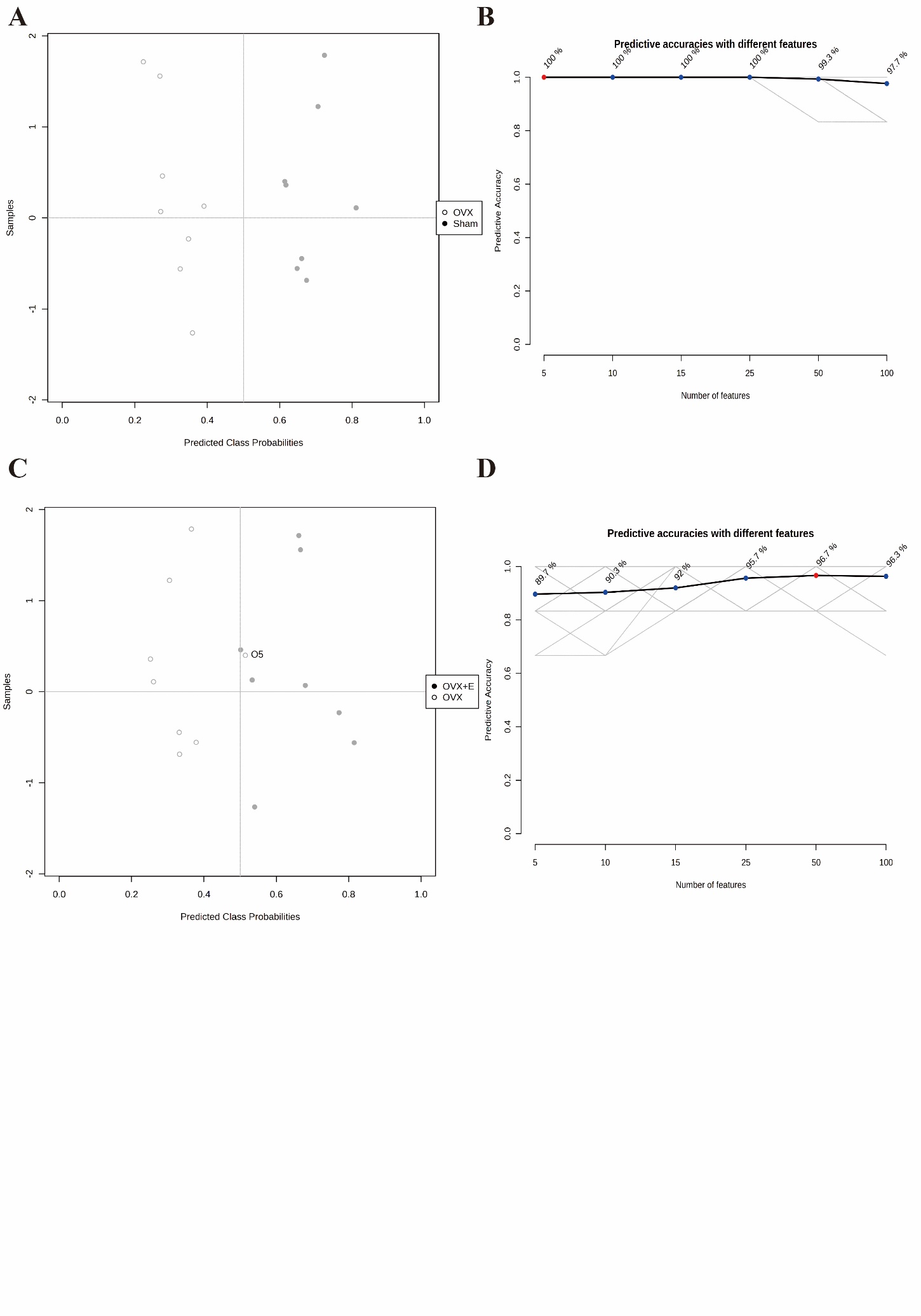
Figure S6：Misclassification diagram of subgroup analysis. (A) The misclassification plot between the sham and OVX group. (B) The ROC prediction accuracy plot between the sham and OVX group. (C) The misclassification plot between OVX group and OVX + E group. (D) The ROC prediction accuracy plot between OVX group and OVX + E group.

Table S1: Summary of me-tabolites detected based on metabolomics; Table S2: Summary of differential metabolites based on ANOVA; Table S3: SAM analysis of differential metabolite based on ANOVA; Table S4: Pearson correlation table of metabolites; Table S5: Pearson correlation table of samples; Table S6: VIP score of differential metabolites based on PLS-DA; Table S7: Cross validation table based on PLS-DA; Table S8: Classification feature scoring based on RF model; Table S9: Differential metabolites between sham group and OVX group based on t-test; Table S10: VIP score of differential metab-olites between sham group and OVX group based on OPLS-DA model; Table S11: VIP score of differential metabolites between OVX group and OVX + E group based on OPLS-DA model; Table S12: Differential metabolite analysis between subgroups based on EBAM method; Table S13: Analysis of differential metabolites between subgroups based on SAM method; Table S14: Ex-pression patterns of OVX related metabolites; Table S15: Expression patterns of metabolites re-lated to estrogen supplementation; Table S16: Expression patterns of metabolites associated with increased estrogen content; Table S17: Expression patterns of lysoprc (15:0) - related metabolites; Table S18: Biomarker screening based on classic uniform ROC curve analysis; Table S19: Bi-omarker screening based on multivariate ROC curve based exploration analysis; Table S20: Av-erage importance ranking based on multivariate ROC curve; Table S21: The KEGG enrichment analysis results; Table S22: The rat-specific metabolic pathways enrichment analysis results.
